# Supplementary material for: Saccharomyces boulardii (CNCM I-745) improves intestinal damage in sepsis by remodeling intestinal flora structure
Source: Front Cell Infect Microbiol. 2025 Jul 31;15:1602792. doi: 10.3389/fcimb.2025.1602792 (PMC12350329; doi:10.3389/fcimb.2025.1602792)
Supplement: Supplementary file 1 [file DataSheet1.zip › summary/summary/7_Differential_analysis/2_LEfSe_result/Group_BW0_vs_AW0_0.05_3.0/LEfSe_Group_BW0_vs_AW0.abund.pdf]

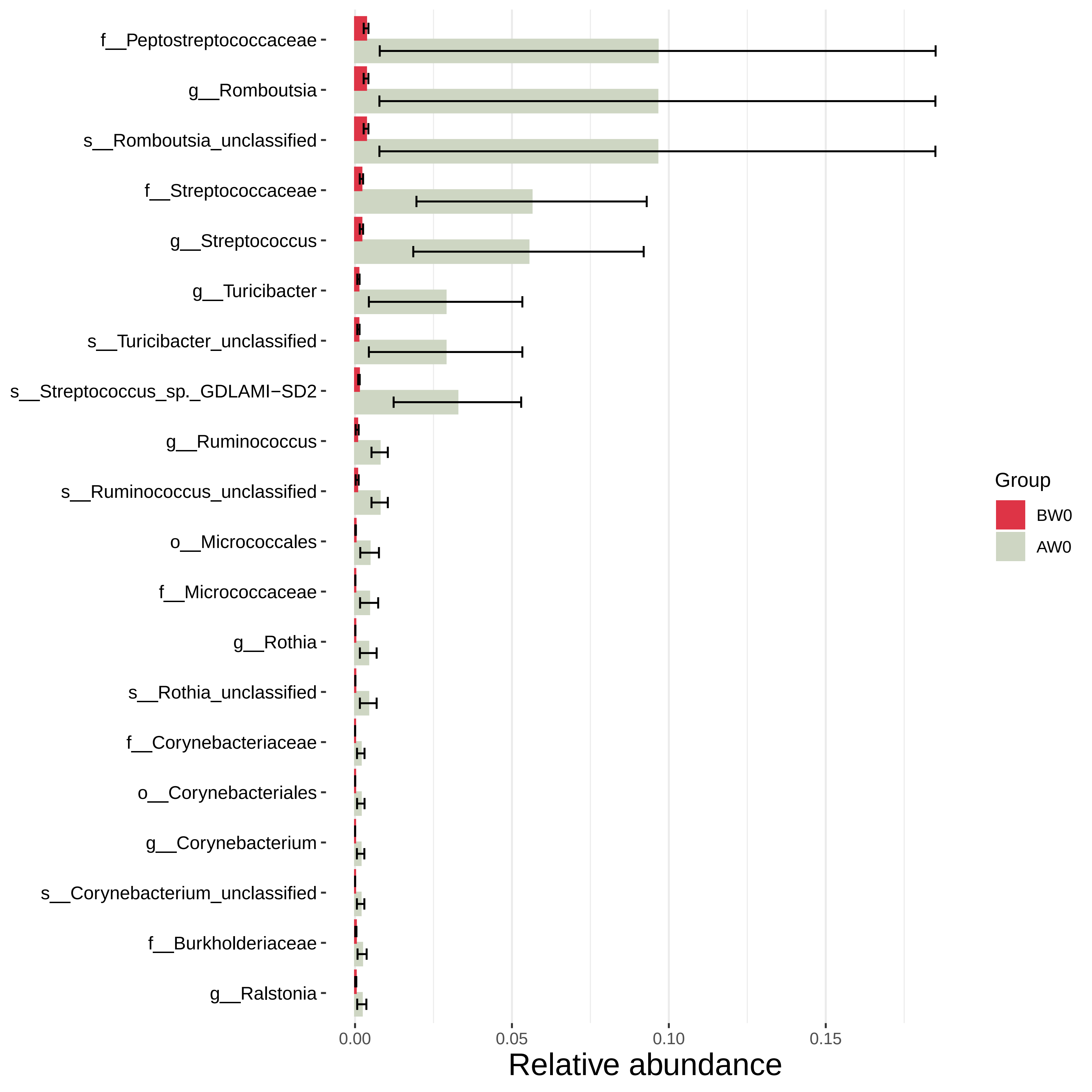
f__Peptostreptococcaceae
g__Romboutsia
s__Romboutsia_unclassified
f__Streptococcaceae
g__Streptococcus
g__Turicibacter
s__Turicibacter_unclassified
s__Streptococcus_sp._GDLAMI−SD2
g__Ruminococcus
s__Ruminococcus_unclassified
o__Micrococcales
f__Micrococcaceae
g__Rothia
s__Rothia_unclassified
f__Corynebacteriaceae
o__Corynebacteriales
g__Corynebacterium
s__Corynebacterium_unclassified
f__Burkholderiaceae
g__Ralstonia
0.00
0.05
0.10
0.15
Relative abundance
Group
BW0
AW0
